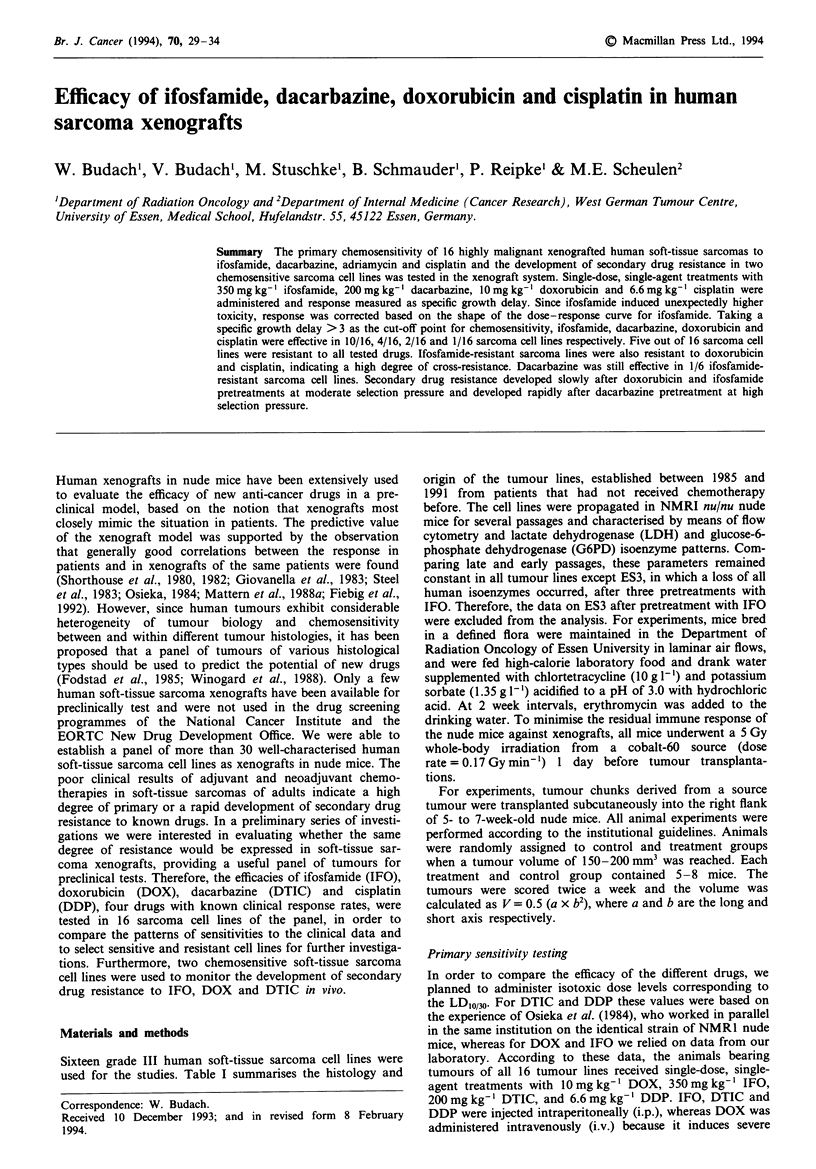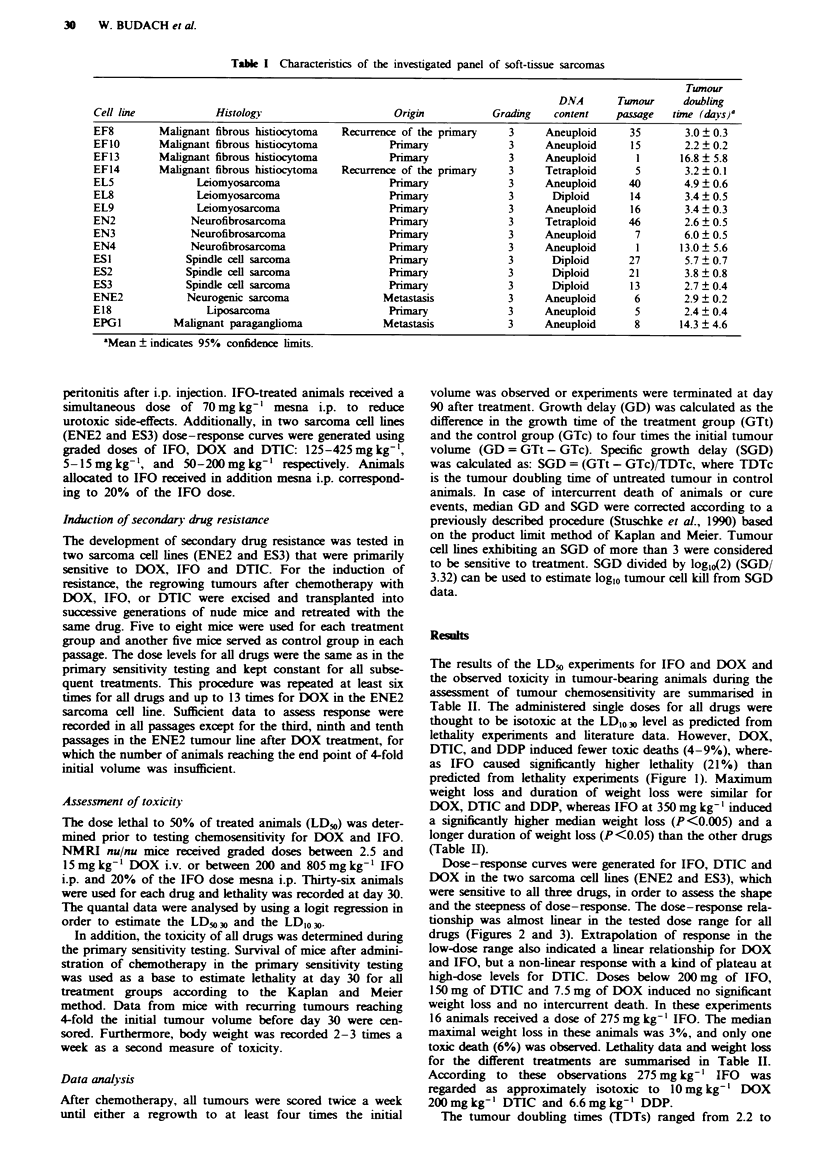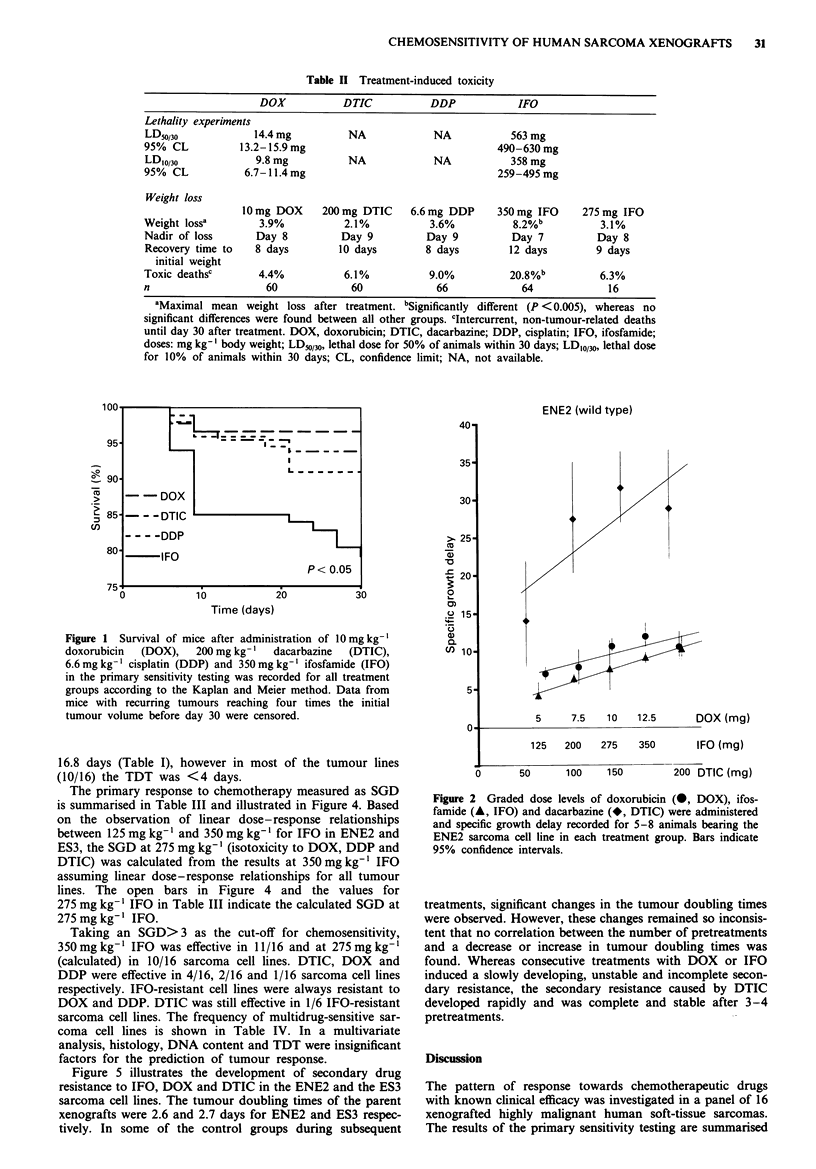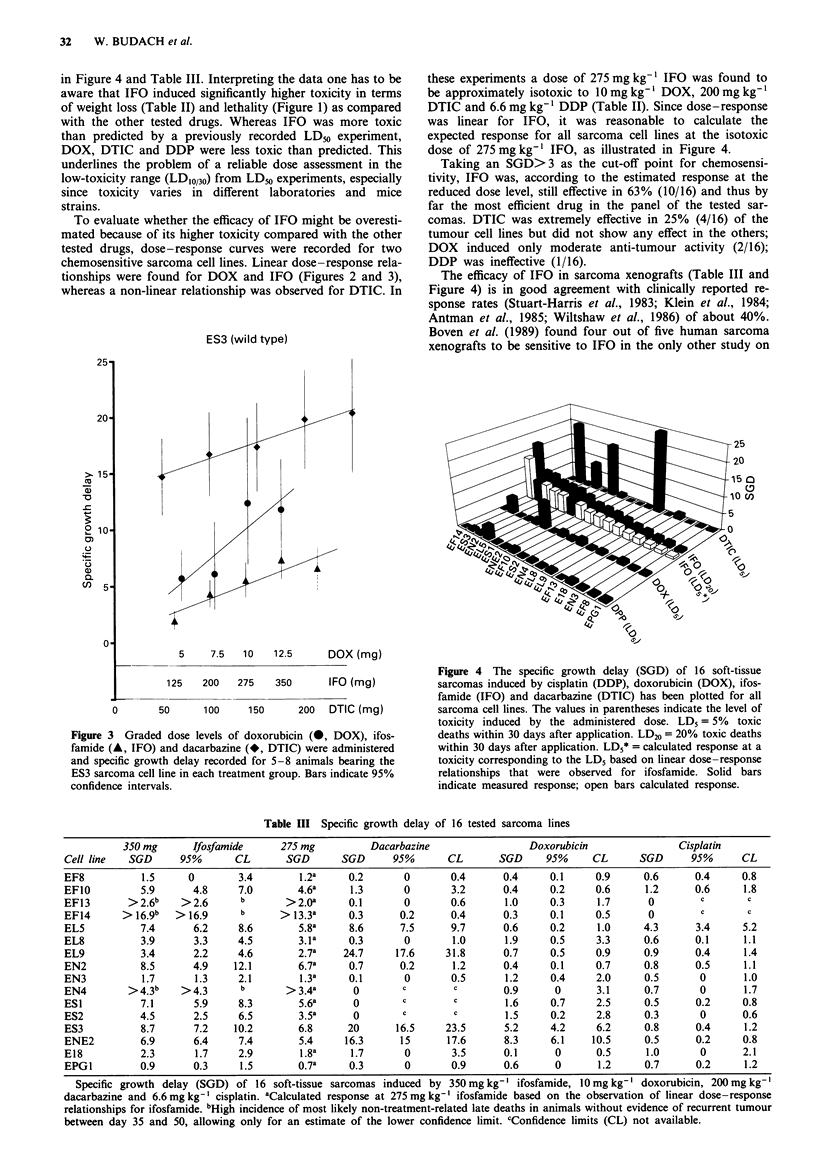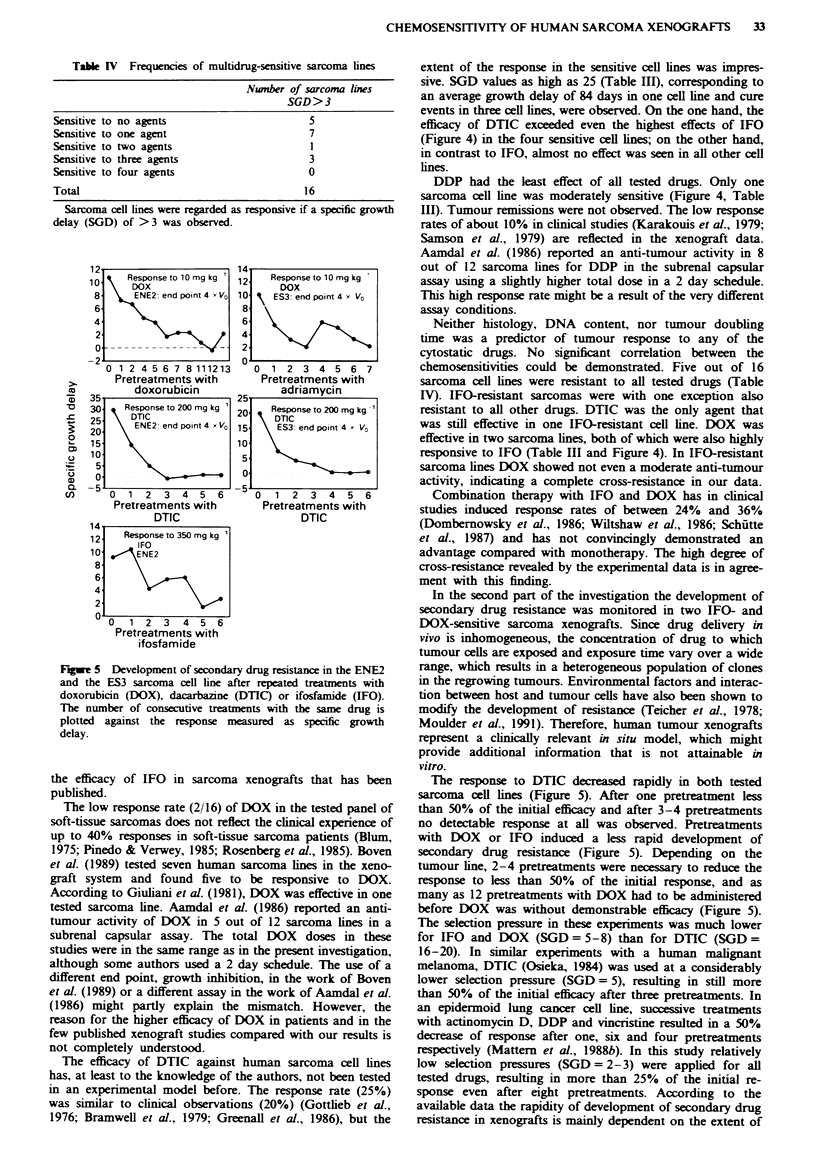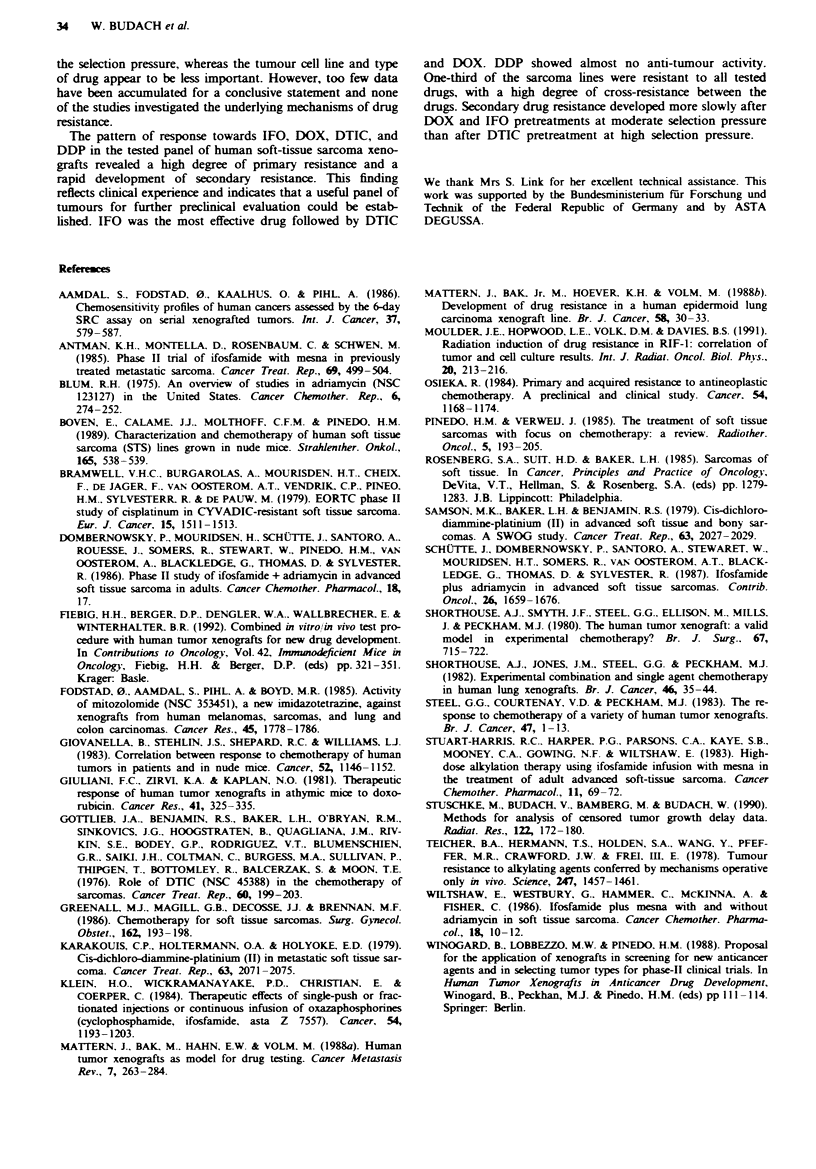# Efficacy of ifosfamide, dacarbazine, doxorubicin and cisplatin in human sarcoma xenografts.

**DOI:** 10.1038/bjc.1994.245

**Published:** 1994-07

**Authors:** W. Budach, V. Budach, M. Stuschke, B. Schmauder, P. Reipke, M. E. Scheulen

**Affiliations:** Department of Radiation Oncology, West German Tumour Centre, University of Essen, Medical School.

## Abstract

The primary chemosensitivity of 16 highly malignant xenografted human soft-tissue sarcomas to ifosfamide, dacarbazine, adriamycin and cisplatin and the development of secondary drug resistance in two chemosensitive sarcoma cell lines was tested in the xenograft system. Single-dose, single-agent treatments with 350 mg kg-1 ifosfamide, 200 mg kg-1 dacarbazine, 10 mg kg-1 doxorubicin and 6.6 mg kg-1 cisplatin were administered and response measured as specific growth delay. Since ifosfamide induced unexpectedly higher toxicity, response was corrected based on the shape of the dose-response curve for ifosfamide. Taking a specific growth delay > 3 as the cut-off point for chemosensitivity, ifosfamide, dacarbazine, doxorubicin and cisplatin were effective in 10/16, 4/16, 2/16 and 1/16 sarcoma cell lines respectively. Five out of 16 sarcoma cell lines were resistant to all tested drugs. Ifosfamide-resistant sarcoma lines were also resistant to doxorubicin and cisplatin, indicating a high degree of cross-resistance. Dacarbazine was still effective in 1/6 ifosfamide-resistant sarcoma cell lines. Secondary drug resistance developed slowly after doxorubicin and ifosfamide pretreatments at moderate selection pressure and developed rapidly after dacarbazine pretreatment at high selection pressure.


*Br. J. Cancer* (1994), **70**, 29–34 © Macmillan Press Ltd., 1994

### Efficacy of ifosfamide, dacarbazine, doxorubicin and cisplatin in human sarcoma xenografts

W. Budach[1], V. Budach[1], M. Stuschke[1], B. Schmauder[1], P. Reipke[1] & M.E. Scheulen[2]

*[1]Department of Radiation Oncology and [2]Department of Internal Medicine (Cancer Research), West German Tumour Centre, University of Essen, Medical School, Hufelandstr. 55, 45122 Essen, Germany.*

**Summary** The primary chemosensitivity of 16 highly malignant xenografted human soft-tissue sarcomas to ifosfamide, dacarbazine, adriamycin and cisplatin and the development of secondary drug resistance in two chemosensitive sarcoma cell lines was tested in the xenograft system. Single-dose, single-agent treatments with 350 mg kg$^{-1}$ ifosfamide, 200 mg kg$^{-1}$ dacarbazine, 10 mg kg$^{-1}$ doxorubicin and 6.6 mg kg$^{-1}$ cisplatin were administered and response measured as specific growth delay. Since ifosfamide induced unexpectedly higher toxicity, response was corrected based on the shape of the dose–response curve for ifosfamide. Taking a specific growth delay $>3$ as the cut-off point for chemosensitivity, ifosfamide, dacarbazine, doxorubicin and cisplatin were effective in 10/16, 4/16, 2/16 and 1/16 sarcoma cell lines respectively. Five out of 16 sarcoma cell lines were resistant to all tested drugs. Ifosfamide-resistant sarcoma lines were also resistant to doxorubicin and cisplatin, indicating a high degree of cross-resistance. Dacarbazine was still effective in 1/6 ifosfamide-resistant sarcoma cell lines. Secondary drug resistance developed slowly after doxorubicin and ifosfamide pretreatments at moderate selection pressure and developed rapidly after dacarbazine pretreatment at high selection pressure.

Human xenografts in nude mice have been extensively used to evaluate the efficacy of new anti-cancer drugs in a preclinical model, based on the notion that xenografts most closely mimic the situation in patients. The predictive value of the xenograft model was supported by the observation that generally good correlations between the response in patients and in xenografts of the same patients were found (Shorthouse *et al.*, 1980, 1982; Giovanella *et al.*, 1983; Steel *et al.*, 1983; Osieka, 1984; Mattern *et al.*, 1988*a*; Fiebig *et al.*, 1992). However, since human tumours exhibit considerable heterogeneity of tumour biology and chemosensitivity between and within different tumour histologies, it has been proposed that a panel of tumours of various histological types should be used to predict the potential of new drugs (Fodstad *et al.*, 1985; Winogard *et al.*, 1988). Only a few human soft-tissue sarcoma xenografts have been available for preclinically test and were not used in the drug screening programmes of the National Cancer Institute and the EORTC New Drug Development Office. We were able to establish a panel of more than 30 well-characterised human soft-tissue sarcoma cell lines as xenografts in nude mice. The poor clinical results of adjuvant and neoadjuvant chemotherapies in soft-tissue sarcomas of adults indicate a high degree of primary or a rapid development of secondary drug resistance to known drugs. In a preliminary series of investigations we were interested in evaluating whether the same degree of resistance would be expressed in soft-tissue sarcoma xenografts, providing a useful panel of tumours for preclinical tests. Therefore, the efficacies of ifosfamide (IFO), doxorubicin (DOX), dacarbazine (DTIC) and cisplatin (DDP), four drugs with known clinical response rates, were tested in 16 sarcoma cell lines of the panel, in order to compare the patterns of sensitivities to the clinical data and to select sensitive and resistant cell lines for further investigations. Furthermore, two chemosensitive soft-tissue sarcoma cell lines were used to monitor the development of secondary drug resistance to IFO, DOX and DTIC *in vivo*.

### Materials and methods

Sixteen grade III human soft-tissue sarcoma cell lines were used for the studies. Table I summarises the histology and origin of the tumour lines, established between 1985 and 1991 from patients that had not received chemotherapy before. The cell lines were propagated in NMRI *nu*/*nu* nude mice for several passages and characterised by means of flow cytometry and lactate dehydrogenase (LDH) and glucose-6-phosphate dehydrogenase (G6PD) isoenzyme patterns. Comparing late and early passages, these parameters remained constant in all tumour lines except ES3, in which a loss of all human isoenzymes occurred, after three pretreatments with IFO. Therefore, the data on ES3 after pretreatment with IFO were excluded from the analysis. For experiments, mice bred in a defined flora were maintained in the Department of Radiation Oncology of Essen University in laminar air flows, and were fed high-calorie laboratory food and drank water supplemented with chlortetracycline (10 g l$^{-1}$) and potassium sorbate (1.35 g l$^{-1}$) acidified to a pH of 3.0 with hydrochloric acid. At 2 week intervals, erythromycin was added to the drinking water. To minimise the residual immune response of the nude mice against xenografts, all mice underwent a 5 Gy whole-body irradiation from a cobalt-60 source (dose rate = 0.17 Gy min$^{-1}$) 1 day before tumour transplantations.

For experiments, tumour chunks derived from a source tumour were transplanted subcutaneously into the right flank of 5- to 7-week-old nude mice. All animal experiments were performed according to the institutional guidelines. Animals were randomly assigned to control and treatment groups when a tumour volume of 150–200 mm$^3$ was reached. Each treatment and control group contained 5–8 mice. The tumours were scored twice a week and the volume was calculated as $V = 0.5\ (a \times b^2)$, where $a$ and $b$ are the long and short axis respectively.

### *Primary sensitivity testing*

In order to compare the efficacy of the different drugs, we planned to administer isotoxic dose levels corresponding to the $LD_{10/30}$. For DTIC and DDP these values were based on the experience of Osieka *et al.* (1984), who worked in parallel in the same institution on the identical strain of NMR1 nude mice, whereas for DOX and IFO we relied on data from our laboratory. According to these data, the animals bearing tumours of all 16 tumour lines received single-dose, single-agent treatments with 10 mg kg$^{-1}$ DOX, 350 mg kg$^{-1}$ IFO, 200 mg kg$^{-1}$ DTIC, and 6.6 mg kg$^{-1}$ DDP. IFO, DTIC and DDP were injected intraperitoneally (i.p.), whereas DOX was administered intravenously (i.v.) because it induces severe

Correspondence: W. Budach.
Received 10 December 1993; and in revised form 8 February 1994.

**Table I** Characteristics of the investigated panel of soft-tissue sarcomas

| Cell line | Histology | Origin | Grading | DNA content | Tumour passage | Tumour doubling time (days)[a] |
|---|---|---|---|---|---|---|
| EF8 | Malignant fibrous histiocytoma | Recurrence of the primary | 3 | Aneuploid | 35 | 3.0 ± 0.3 |
| EF10 | Malignant fibrous histiocytoma | Primary | 3 | Aneuploid | 15 | 2.2 ± 0.2 |
| EF13 | Malignant fibrous histiocytoma | Primary | 3 | Aneuploid | 1 | 16.8 ± 5.8 |
| EF14 | Malignant fibrous histiocytoma | Recurrence of the primary | 3 | Tetraploid | 5 | 3.2 ± 0.1 |
| EL5 | Leiomyosarcoma | Primary | 3 | Aneuploid | 40 | 4.9 ± 0.6 |
| EL8 | Leiomyosarcoma | Primary | 3 | Diploid | 14 | 3.4 ± 0.5 |
| EL9 | Leiomyosarcoma | Primary | 3 | Aneuploid | 16 | 3.4 ± 0.3 |
| EN2 | Neurofibrosarcoma | Primary | 3 | Tetraploid | 46 | 2.6 ± 0.5 |
| EN3 | Neurofibrosarcoma | Primary | 3 | Aneuploid | 7 | 6.0 ± 0.5 |
| EN4 | Neurofibrosarcoma | Primary | 3 | Aneuploid | 1 | 13.0 ± 5.6 |
| ES1 | Spindle cell sarcoma | Primary | 3 | Diploid | 27 | 5.7 ± 0.7 |
| ES2 | Spindle cell sarcoma | Primary | 3 | Diploid | 21 | 3.8 ± 0.8 |
| ES3 | Spindle cell sarcoma | Primary | 3 | Diploid | 13 | 2.7 ± 0.4 |
| ENE2 | Neurogenic sarcoma | Metastasis | 3 | Aneuploid | 6 | 2.9 ± 0.2 |
| E18 | Liposarcoma | Primary | 3 | Aneuploid | 5 | 2.4 ± 0.4 |
| EPG1 | Malignant paraganglioma | Metastasis | 3 | Aneuploid | 8 | 14.3 ± 4.6 |

[a]Mean ± indicates 95% confidence limits.

peritonitis after i.p. injection. IFO-treated animals received a simultaneous dose of 70 mg kg$^{-1}$ mesna i.p. to reduce urotoxic side-effects. Additionally, in two sarcoma cell lines (ENE2 and ES3) dose–response curves were generated using graded doses of IFO, DOX and DTIC: 125–425 mg kg$^{-1}$, 5–15 mg kg$^{-1}$, and 50–200 mg kg$^{-1}$ respectively. Animals allocated to IFO received in addition mesna i.p. corresponding to 20% of the IFO dose.

*Induction of secondary drug resistance*

The development of secondary drug resistance was tested in two sarcoma cell lines (ENE2 and ES3) that were primarily sensitive to DOX, IFO and DTIC. For the induction of resistance, the regrowing tumours after chemotherapy with DOX, IFO, or DTIC were excised and transplanted into successive generations of nude mice and retreated with the same drug. Five to eight mice were used for each treatment group and another five mice served as control group in each passage. The dose levels for all drugs were the same as in the primary sensitivity testing and kept constant for all subsequent treatments. This procedure was repeated at least six times for all drugs and up to 13 times for DOX in the ENE2 sarcoma cell line. Sufficient data to assess response were recorded in all passages except for the third, ninth and tenth passages in the ENE2 tumour line after DOX treatment, for which the number of animals reaching the end point of 4-fold initial volume was insufficient.

*Assessment of toxicity*

The dose lethal to 50% of treated animals ($LD_{50}$) was determined prior to testing chemosensitivity for DOX and IFO. NMRI *nu/nu* mice received graded doses between 2.5 and 15 mg kg$^{-1}$ DOX i.v. or between 200 and 805 mg kg$^{-1}$ IFO i.p. and 20% of the IFO dose mesna i.p. Thirty-six animals were used for each drug and lethality was recorded at day 30. The quantal data were analysed by using a logit regression in order to estimate the $LD_{50\,30}$ and the $LD_{10\,30}$.

In addition, the toxicity of all drugs was determined during the primary sensitivity testing. Survival of mice after administration of chemotherapy in the primary sensitivity testing was used as a base to estimate lethality at day 30 for all treatment groups according to the Kaplan and Meier method. Data from mice with recurring tumours reaching 4-fold the initial tumour volume before day 30 were censored. Furthermore, body weight was recorded 2–3 times a week as a second measure of toxicity.

*Data analysis*

After chemotherapy, all tumours were scored twice a week until either a regrowth to at least four times the initial volume was observed or experiments were terminated at day 90 after treatment. Growth delay (GD) was calculated as the difference in the growth time of the treatment group (GTt) and the control group (GTc) to four times the initial tumour volume (GD = GTt − GTc). Specific growth delay (SGD) was calculated as: SGD = (GTt − GTc)/TDTc, where TDTc is the tumour doubling time of untreated tumour in control animals. In case of intercurrent death of animals or cure events, median GD and SGD were corrected according to a previously described procedure (Stuschke *et al*., 1990) based on the product limit method of Kaplan and Meier. Tumour cell lines exhibiting an SGD of more than 3 were considered to be sensitive to treatment. SGD divided by $\log_{10}(2)$ (SGD/3.32) can be used to estimate $\log_{10}$ tumour cell kill from SGD data.

### Results

The results of the $LD_{50}$ experiments for IFO and DOX and the observed toxicity in tumour-bearing animals during the assessment of tumour chemosensitivity are summarised in Table II. The administered single doses for all drugs were thought to be isotoxic at the $LD_{10\,30}$ level as predicted from lethality experiments and literature data. However, DOX, DTIC, and DDP induced fewer toxic deaths (4–9%), whereas IFO caused significantly higher lethality (21%) than predicted from lethality experiments (Figure 1). Maximum weight loss and duration of weight loss were similar for DOX, DTIC and DDP, whereas IFO at 350 mg kg$^{-1}$ induced a significantly higher median weight loss ($P<0.005$) and a longer duration of weight loss ($P<0.05$) than the other drugs (Table II).

Dose–response curves were generated for IFO, DTIC and DOX in the two sarcoma cell lines (ENE2 and ES3), which were sensitive to all three drugs, in order to assess the shape and the steepness of dose–response. The dose–response relationship was almost linear in the tested dose range for all drugs (Figures 2 and 3). Extrapolation of response in the low-dose range also indicated a linear relationship for DOX and IFO, but a non-linear response with a kind of plateau at high-dose levels for DTIC. Doses below 200 mg of IFO, 150 mg of DTIC and 7.5 mg of DOX induced no significant weight loss and no intercurrent death. In these experiments 16 animals received a dose of 275 mg kg$^{-1}$ IFO. The median maximal weight loss in these animals was 3%, and only one toxic death (6%) was observed. Lethality data and weight loss for the different treatments are summarised in Table II. According to these observations 275 mg kg$^{-1}$ IFO was regarded as approximately isotoxic to 10 mg kg$^{-1}$ DOX 200 mg kg$^{-1}$ DTIC and 6.6 mg kg$^{-1}$ DDP.

The tumour doubling times (TDTs) ranged from 2.2 to

**Table II** Treatment-induced toxicity

| | *DOX* | *DTIC* | *DDP* | *IFO* | |
|---|---|---|---|---|---|
| *Lethality experiments* | | | | | |
| $LD_{50/30}$ | 14.4 mg | NA | NA | 563 mg | |
| 95% CL | 13.2–15.9 mg | | | 490–630 mg | |
| $LD_{10/30}$ | 9.8 mg | NA | NA | 358 mg | |
| 95% CL | 6.7–11.4 mg | | | 259–495 mg | |
| *Weight loss* | | | | | |
| | 10 mg DOX | 200 mg DTIC | 6.6 mg DDP | 350 mg IFO | 275 mg IFO |
| Weight loss[a] | 3.9% | 2.1% | 3.6% | 8.2%[b] | 3.1% |
| Nadir of loss | Day 8 | Day 9 | Day 9 | Day 7 | Day 8 |
| Recovery time to initial weight | 8 days | 10 days | 8 days | 12 days | 9 days |
| Toxic deaths[c] | 4.4% | 6.1% | 9.0% | 20.8%[b] | 6.3% |
| *n* | 60 | 60 | 66 | 64 | 16 |

[a]Maximal mean weight loss after treatment. [b]Significantly different ($P < 0.005$), whereas no significant differences were found between all other groups. [c]Intercurrent, non-tumour-related deaths until day 30 after treatment. DOX, doxorubicin; DTIC, dacarbazine; DDP, cisplatin; IFO, ifosfamide; doses: mg $kg^{-1}$ body weight; $LD_{50/30}$, lethal dose for 50% of animals within 30 days; $LD_{10/30}$, lethal dose for 10% of animals within 30 days; CL, confidence limit; NA, not available.

**Figure 1** Survival of mice after administration of 10 mg $kg^{-1}$ doxorubicin (DOX), 200 mg $kg^{-1}$ dacarbazine (DTIC), 6.6 mg $kg^{-1}$ cisplatin (DDP) and 350 mg $kg^{-1}$ ifosfamide (IFO) in the primary sensitivity testing was recorded for all treatment groups according to the Kaplan and Meier method. Data from mice with recurring tumours reaching four times the initial tumour volume before day 30 were censored.

**Figure 2** Graded dose levels of doxorubicin (●, DOX), ifosfamide (▲, IFO) and dacarbazine (◆, DTIC) were administered and specific growth delay recorded for 5–8 animals bearing the ENE2 sarcoma cell line in each treatment group. Bars indicate 95% confidence intervals.

16.8 days (Table I), however in most of the tumour lines (10/16) the TDT was <4 days.

The primary response to chemotherapy measured as SGD is summarised in Table III and illustrated in Figure 4. Based on the observation of linear dose–response relationships between 125 mg $kg^{-1}$ and 350 mg $kg^{-1}$ for IFO in ENE2 and ES3, the SGD at 275 mg $kg^{-1}$ (isotoxicity to DOX, DDP and DTIC) was calculated from the results at 350 mg $kg^{-1}$ IFO assuming linear dose–response relationships for all tumour lines. The open bars in Figure 4 and the values for 275 mg $kg^{-1}$ IFO in Table III indicate the calculated SGD at 275 mg $kg^{-1}$ IFO.

Taking an SGD>3 as the cut-off for chemosensitivity, 350 mg $kg^{-1}$ IFO was effective in 11/16 and at 275 mg $kg^{-1}$ (calculated) in 10/16 sarcoma cell lines. DTIC, DOX and DDP were effective in 4/16, 2/16 and 1/16 sarcoma cell lines respectively. IFO-resistant cell lines were always resistant to DOX and DDP. DTIC was still effective in 1/6 IFO-resistant sarcoma cell lines. The frequency of multidrug-sensitive sarcoma cell lines is shown in Table IV. In a multivariate analysis, histology, DNA content and TDT were insignificant factors for the prediction of tumour response.

Figure 5 illustrates the development of secondary drug resistance to IFO, DOX and DTIC in the ENE2 and the ES3 sarcoma cell lines. The tumour doubling times of the parent xenografts were 2.6 and 2.7 days for ENE2 and ES3 respectively. In some of the control groups during subsequent treatments, significant changes in the tumour doubling times were observed. However, these changes remained so inconsistent that no correlation between the number of pretreatments and a decrease or increase in tumour doubling times was found. Whereas consecutive treatments with DOX or IFO induced a slowly developing, unstable and incomplete secondary resistance, the secondary resistance caused by DTIC developed rapidly and was complete and stable after 3–4 pretreatments.

### Discussion

The pattern of response towards chemotherapeutic drugs with known clinical efficacy was investigated in a panel of 16 xenografted highly malignant human soft-tissue sarcomas. The results of the primary sensitivity testing are summarised in Figure 4 and Table III. Interpreting the data one has to be aware that IFO induced significantly higher toxicity in terms of weight loss (Table II) and lethality (Figure 1) as compared with the other tested drugs. Whereas IFO was more toxic than predicted by a previously recorded $LD_{50}$ experiment, DOX, DTIC and DDP were less toxic than predicted. This underlines the problem of a reliable dose assessment in the low-toxicity range ($LD_{10/30}$) from $LD_{50}$ experiments, especially since toxicity varies in different laboratories and mice strains.

To evaluate whether the efficacy of IFO might be overestimated because of its higher toxicity compared with the other tested drugs, dose–response curves were recorded for two chemosensitive sarcoma cell lines. Linear dose–response relationships were found for DOX and IFO (Figures 2 and 3), whereas a non-linear relationship was observed for DTIC. In these experiments a dose of 275 mg $kg^{-1}$ IFO was found to be approximately isotoxic to 10 mg $kg^{-1}$ DOX, 200 mg $kg^{-1}$ DTIC and 6.6 mg $kg^{-1}$ DDP (Table II). Since dose–response was linear for IFO, it was reasonable to calculate the expected response for all sarcoma cell lines at the isotoxic dose of 275 mg $kg^{-1}$ IFO, as illustrated in Figure 4.

Taking an SGD > 3 as the cut-off point for chemosensitivity, IFO was, according to the estimated response at the reduced dose level, still effective in 63% (10/16) and thus by far the most efficient drug in the panel of the tested sarcomas. DTIC was extremely effective in 25% (4/16) of the tumour cell lines but did not show any effect in the others; DOX induced only moderate anti-tumour activity (2/16); DDP was ineffective (1/16).

The efficacy of IFO in sarcoma xenografts (Table III and Figure 4) is in good agreement with clinically reported response rates (Stuart-Harris et al., 1983; Klein et al., 1984; Antman et al., 1985; Wiltshaw et al., 1986) of about 40%. Boven et al. (1989) found four out of five human sarcoma xenografts to be sensitive to IFO in the only other study on

**Figure 3** Graded dose levels of doxorubicin (●, DOX), ifosfamide (▲, IFO) and dacarbazine (◆, DTIC) were administered and specific growth delay recorded for 5–8 animals bearing the ES3 sarcoma cell line in each treatment group. Bars indicate 95% confidence intervals.

**Figure 4** The specific growth delay (SGD) of 16 soft-tissue sarcomas induced by cisplatin (DDP), doxorubicin (DOX), ifosfamide (IFO) and dacarbazine (DTIC) has been plotted for all sarcoma cell lines. The values in parentheses indicate the level of toxicity induced by the administered dose. $LD_5$ = 5% toxic deaths within 30 days after application. $LD_{20}$ = 20% toxic deaths within 30 days after application. $LD_5$* = calculated response at a toxicity corresponding to the $LD_5$ based on linear dose–response relationships that were observed for ifosfamide. Solid bars indicate measured response; open bars calculated response.

**Table III** Specific growth delay of 16 tested sarcoma lines

| Cell line | 350 mg SGD | Ifosfamide 95% | CL | 275 mg SGD | Dacarbazine SGD | 95% | CL | Doxorubicin SGD | 95% | CL | Cisplatin SGD | 95% | CL |
|---|---|---|---|---|---|---|---|---|---|---|---|---|---|
| EF8 | 1.5 | 0 | 3.4 | 1.2[a] | 0.2 | 0 | 0.4 | 0.4 | 0.1 | 0.9 | 0.6 | 0.4 | 0.8 |
| EF10 | 5.9 | 4.8 | 7.0 | 4.6[a] | 1.3 | 0 | 3.2 | 0.4 | 0.2 | 0.6 | 1.2 | 0.6 | 1.8 |
| EF13 | >2.6[b] | >2.6 | [b] | >2.0[a] | 0.1 | 0 | 0.6 | 1.0 | 0.3 | 1.7 | 0 | [c] | [c] |
| EF14 | >16.9[b] | >16.9 | [b] | >13.3[a] | 0.3 | 0.2 | 0.4 | 0.3 | 0.1 | 0.5 | 0 | [c] | [c] |
| EL5 | 7.4 | 6.2 | 8.6 | 5.8[a] | 8.6 | 7.5 | 9.7 | 0.6 | 0.2 | 1.0 | 4.3 | 3.4 | 5.2 |
| EL8 | 3.9 | 3.3 | 4.5 | 3.1[a] | 0.3 | 0 | 1.0 | 1.9 | 0.5 | 3.3 | 0.6 | 0.1 | 1.1 |
| EL9 | 3.4 | 2.2 | 4.6 | 2.7[a] | 24.7 | 17.6 | 31.8 | 0.7 | 0.5 | 0.9 | 0.9 | 0.4 | 1.4 |
| EN2 | 8.5 | 4.9 | 12.1 | 6.7[a] | 0.7 | 0.2 | 1.2 | 0.4 | 0.1 | 0.7 | 0.8 | 0.5 | 1.1 |
| EN3 | 1.7 | 1.3 | 2.1 | 1.3[a] | 0.1 | 0 | 0.5 | 1.2 | 0.4 | 2.0 | 0.5 | 0 | 1.0 |
| EN4 | >4.3[b] | >4.3 | [b] | >3.4[a] | 0 | [c] | [c] | 0.9 | 0 | 3.1 | 0.7 | 0 | 1.7 |
| ES1 | 7.1 | 5.9 | 8.3 | 5.6[a] | 0 | [c] | [c] | 1.6 | 0.7 | 2.5 | 0.5 | 0.2 | 0.8 |
| ES2 | 4.5 | 2.5 | 6.5 | 3.5[a] | 0 | [c] | [c] | 1.5 | 0.2 | 2.8 | 0.3 | 0 | 0.6 |
| ES3 | 8.7 | 7.2 | 10.2 | 6.8 | 20 | 16.5 | 23.5 | 5.2 | 4.2 | 6.2 | 0.8 | 0.4 | 1.2 |
| ENE2 | 6.9 | 6.4 | 7.4 | 5.4 | 16.3 | 15 | 17.6 | 8.3 | 6.1 | 10.5 | 0.5 | 0.2 | 0.8 |
| E18 | 2.3 | 1.7 | 2.9 | 1.8[a] | 1.7 | 0 | 3.5 | 0.1 | 0 | 0.5 | 1.0 | 0 | 2.1 |
| EPG1 | 0.9 | 0.3 | 1.5 | 0.7[a] | 0.3 | 0 | 0.9 | 0.6 | 0 | 1.2 | 0.7 | 0.2 | 1.2 |

Specific growth delay (SGD) of 16 soft-tissue sarcomas induced by 350 mg $kg^{-1}$ ifosfamide, 10 mg $kg^{-1}$ doxorubicin, 200 mg $kg^{-1}$ dacarbazine and 6.6 mg $kg^{-1}$ cisplatin. [a]Calculated response at 275 mg $kg^{-1}$ ifosfamide based on the observation of linear dose–response relationships for ifosfamide. [b]High incidence of most likely non-treatment-related late deaths in animals without evidence of recurrent tumour between day 35 and 50, allowing only for an estimate of the lower confidence limit. [c]Confidence limits (CL) not available.

**Table IV** Frequencies of multidrug-sensitive sarcoma lines

| | *Number of sarcoma lines SGD>3* |
|---|---|
| Sensitive to no agents | 5 |
| Sensitive to one agent | 7 |
| Sensitive to two agents | 1 |
| Sensitive to three agents | 3 |
| Sensitive to four agents | 0 |
| Total | 16 |

Sarcoma cell lines were regarded as responsive if a specific growth delay (SGD) of >3 was observed.

**Figure 5** Development of secondary drug resistance in the ENE2 and the ES3 sarcoma cell line after repeated treatments with doxorubicin (DOX), dacarbazine (DTIC) or ifosfamide (IFO). The number of consecutive treatments with the same drug is plotted against the response measured as specific growth delay.

the efficacy of IFO in sarcoma xenografts that has been published.

The low response rate (2/16) of DOX in the tested panel of soft-tissue sarcomas does not reflect the clinical experience of up to 40% responses in soft-tissue sarcoma patients (Blum, 1975; Pinedo & Verwey, 1985; Rosenberg *et al.*, 1985). Boven *et al.* (1989) tested seven human sarcoma lines in the xenograft system and found five to be responsive to DOX. According to Giuliani *et al.* (1981), DOX was effective in one tested sarcoma line. Aamdal *et al.* (1986) reported an anti-tumour activity of DOX in 5 out of 12 sarcoma lines in a subrenal capsular assay. The total DOX doses in these studies were in the same range as in the present investigation, although some authors used a 2 day schedule. The use of a different end point, growth inhibition, in the work of Boven *et al.* (1989) or a different assay in the work of Aamdal *et al.* (1986) might partly explain the mismatch. However, the reason for the higher efficacy of DOX in patients and in the few published xenograft studies compared with our results is not completely understood.

The efficacy of DTIC against human sarcoma cell lines has, at least to the knowledge of the authors, not been tested in an experimental model before. The response rate (25%) was similar to clinical observations (20%) (Gottlieb *et al.*, 1976; Bramwell *et al.*, 1979; Greenall *et al.*, 1986), but the extent of the response in the sensitive cell lines was impressive. SGD values as high as 25 (Table III), corresponding to an average growth delay of 84 days in one cell line and cure events in three cell lines, were observed. On the one hand, the efficacy of DTIC exceeded even the highest effects of IFO (Figure 4) in the four sensitive cell lines; on the other hand, in contrast to IFO, almost no effect was seen in all other cell lines.

DDP had the least effect of all tested drugs. Only one sarcoma cell line was moderately sensitive (Figure 4, Table III). Tumour remissions were not observed. The low response rates of about 10% in clinical studies (Karakouis *et al.*, 1979; Samson *et al.*, 1979) are reflected in the xenograft data. Aamdal *et al.* (1986) reported an anti-tumour activity in 8 out of 12 sarcoma lines for DDP in the subrenal capsular assay using a slightly higher total dose in a 2 day schedule. This high response rate might be a result of the very different assay conditions.

Neither histology, DNA content, nor tumour doubling time was a predictor of tumour response to any of the cytostatic drugs. No significant correlation between the chemosensitivities could be demonstrated. Five out of 16 sarcoma cell lines were resistant to all tested drugs (Table IV). IFO-resistant sarcomas were with one exception also resistant to all other drugs. DTIC was the only agent that was still effective in one IFO-resistant cell line. DOX was effective in two sarcoma lines, both of which were also highly responsive to IFO (Table III and Figure 4). In IFO-resistant sarcoma lines DOX showed not even a moderate anti-tumour activity, indicating a complete cross-resistance in our data.

Combination therapy with IFO and DOX has in clinical studies induced response rates of between 24% and 36% (Dombernowsky *et al.*, 1986; Wiltshaw *et al.*, 1986; Schütte *et al.*, 1987) and has not convincingly demonstrated an advantage compared with monotherapy. The high degree of cross-resistance revealed by the experimental data is in agreement with this finding.

In the second part of the investigation the development of secondary drug resistance was monitored in two IFO- and DOX-sensitive sarcoma xenografts. Since drug delivery *in vivo* is inhomogeneous, the concentration of drug to which tumour cells are exposed and exposure time vary over a wide range, which results in a heterogeneous population of clones in the regrowing tumours. Environmental factors and interaction between host and tumour cells have also been shown to modify the development of resistance (Teicher *et al.*, 1978; Moulder *et al.*, 1991). Therefore, human tumour xenografts represent a clinically relevant *in situ* model, which might provide additional information that is not attainable *in vitro*.

The response to DTIC decreased rapidly in both tested sarcoma cell lines (Figure 5). After one pretreatment less than 50% of the initial efficacy and after 3–4 pretreatments no detectable response at all was observed. Pretreatments with DOX or IFO induced a less rapid development of secondary drug resistance (Figure 5). Depending on the tumour line, 2–4 pretreatments were necessary to reduce the response to less than 50% of the initial response, and as many as 12 pretreatments with DOX had to be administered before DOX was without demonstrable efficacy (Figure 5). The selection pressure in these experiments was much lower for IFO and DOX (SGD = 5–8) than for DTIC (SGD = 16–20). In similar experiments with a human malignant melanoma, DTIC (Osieka, 1984) was used at a considerably lower selection pressure (SGD = 5), resulting in still more than 50% of the initial efficacy after three pretreatments. In an epidermoid lung cancer cell line, successive treatments with actinomycin D, DDP and vincristine resulted in a 50% decrease of response after one, six and four pretreatments respectively (Mattern *et al.*, 1988*b*). In this study relatively low selection pressures (SGD = 2–3) were applied for all tested drugs, resulting in more than 25% of the initial response even after eight pretreatments. According to the available data the rapidity of development of secondary drug resistance in xenografts is mainly dependent on the extent of the selection pressure, whereas the tumour cell line and type of drug appear to be less important. However, too few data have been accumulated for a conclusive statement and none of the studies investigated the underlying mechanisms of drug resistance.

The pattern of response towards IFO, DOX, DTIC, and DDP in the tested panel of human soft-tissue sarcoma xenografts revealed a high degree of primary resistance and a rapid development of secondary resistance. This finding reflects clinical experience and indicates that a useful panel of tumours for further preclinical evaluation could be established. IFO was the most effective drug followed by DTIC and DOX. DDP showed almost no anti-tumour activity. One-third of the sarcoma lines were resistant to all tested drugs, with a high degree of cross-resistance between the drugs. Secondary drug resistance developed more slowly after DOX and IFO pretreatments at moderate selection pressure than after DTIC pretreatment at high selection pressure.

We thank Mrs S. Link for her excellent technical assistance. This work was supported by the Bundesministerium für Forschung und Technik of the Federal Republic of Germany and by ASTA DEGUSSA.

### References

AAMDAL, S., FODSTAD, Ø., KAALHUS, O. & PIHL, A. (1986). Chemosensitivity profiles of human cancers assessed by the 6-day SRC assay on serial xenografted tumors. *Int. J. Cancer*, **37**, 579-587.

ANTMAN, K.H., MONTELLA, D., ROSENBAUM, C. & SCHWEN, M. (1985). Phase II trial of ifosfamide with mesna in previously treated metastatic sarcoma. *Cancer Treat. Rep.*, **69**, 499-504.

BLUM, R.H. (1975). An overview of studies in adriamycin (NSC 123127) in the United States. *Cancer Chemother. Rep.*, **6**, 274-252.

BOVEN, E., CALAME, J.J., MOLTHOFF, C.F.M. & PINEDO, H.M. (1989). Characterization and chemotherapy of human soft tissue sarcoma (STS) lines grown in nude mice. *Strahlenther. Onkol.*, **165**, 538-539.

BRAMWELL, V.H.C., BURGAROLAS, A., MOURISDEN, H.T., CHEIX, F., DE JAGER, F., VAN OOSTEROM, A.T., VENDRIK, C.P., PINEO, H.M., SYLVESTERR, R. & DE PAUW, M. (1979). EORTC phase II study of cisplatinum in CYVADIC-resistant soft tissue sarcoma. *Eur. J. Cancer*, **15**, 1511-1513.

DOMBERNOWSKY, P., MOURIDSEN, H., SCHÜTTE, J., SANTORO, A., ROUESSE, J., SOMERS, R., STEWART, W., PINEDO, H.M., VAN OOSTEROM, A., BLACKLEDGE, G., THOMAS, D. & SYLVESTER, R. (1986). Phase II study of ifosfamide + adriamycin in advanced soft tissue sarcoma in adults. *Cancer Chemother. Pharmacol.*, **18**, 17.

FIEBIG, H.H., BERGER, D.P., DENGLER, W.A., WALLBRECHER, E. & WINTERHALTER, B.R. (1992). Combined *in vitro/in vivo* test procedure with human tumor xenografts for new drug development. In *Contributions to Oncology*, Vol. 42, *Immunodeficient Mice in Oncology*, Fiebig, H.H. & Berger, D.P. (eds) pp. 321-351. Krager: Basle.

FODSTAD, Ø., AAMDAL, S., PIHL, A. & BOYD, M.R. (1985). Activity of mitozolomide (NSC 353451), a new imidazotetrazine, against xenografts from human melanomas, sarcomas, and lung and colon carcinomas. *Cancer Res.*, **45**, 1778-1786.

GIOVANELLA, B., STEHLIN, J.S., SHEPARD, R.C. & WILLIAMS, L.J. (1983). Correlation between response to chemotherapy of human tumors in patients and in nude mice. *Cancer*, **52**, 1146-1152.

GIULIANI, F.C., ZIRVI, K.A. & KAPLAN, N.O. (1981). Therapeutic response of human tumor xenografts in athymic mice to doxorubicin. *Cancer Res.*, **41**, 325-335.

GOTTLIEB, J.A., BENJAMIN, R.S., BAKER, L.H., O'BRYAN, R.M., SINKOVICS, J.G., HOOGSTRATEN, B., QUAGLIANA, J.M., RIVKIN, S.E., BODEY, G.P., RODRIGUEZ, V.T., BLUMENSCHIEN, G.R., SAIKI, J.H., COLTMAN, C., BURGESS, M.A., SULLIVAN, P., THIPGEN, T., BOTTOMLEY, R., BALCERZAK, S. & MOON, T.E. (1976). Role of DTIC (NSC 45388) in the chemotherapy of sarcomas. *Cancer Treat. Rep.*, **60**, 199-203.

GREENALL, M.J., MAGILL, G.B., DECOSSE, J.J. & BRENNAN, M.F. (1986). Chemotherapy for soft tissue sarcomas. *Surg. Gynecol. Obstet.*, **162**, 193-198.

KARAKOUIS, C.P., HOLTERMANN, O.A. & HOLYOKE, E.D. (1979). Cis-dichloro-diammine-platinium (II) in metastatic soft tissue sarcoma. *Cancer Treat. Rep.*, **63**, 2071-2075.

KLEIN, H.O., WICKRAMANAYAKE, P.D., CHRISTIAN, E. & COERPER, C. (1984). Therapeutic effects of single-push or fractionated injections or continuous infusion of oxazaphosphorines (cyclophosphamide, ifosfamide, asta Z 7557). *Cancer*, **54**, 1193-1203.

MATTERN, J., BAK, M., HAHN, E.W. & VOLM, M. (1988*a*). Human tumor xenografts as model for drug testing. *Cancer Metastasis Rev.*, **7**, 263-284.

MATTERN, J., BAK, Jr, M., HOEVER, K.H. & VOLM, M. (1988*b*). Development of drug resistance in a human epidermoid lung carcinoma xenograft line. *Br. J. Cancer*, **58**, 30-33.

MOULDER, J.E., HOPWOOD, L.E., VOLK, D.M. & DAVIES, B.S. (1991). Radiation induction of drug resistance in RIF-1: correlation of tumor and cell culture results. *Int. J. Radiat. Oncol. Biol. Phys.*, **20**, 213-216.

OSIEKA, R. (1984). Primary and acquired resistance to antineoplastic chemotherapy. A preclinical and clinical study. *Cancer*, **54**, 1168-1174.

PINEDO, H.M. & VERWEIJ, J. (1985). The treatment of soft tissue sarcomas with focus on chemotherapy: a review. *Radiother. Oncol.*, **5**, 193-205.

ROSENBERG, S.A., SUIT, H.D. & BAKER, L.H. (1985). Sarcomas of soft tissue. In *Cancer, Principles and Practice of Oncology*, DeVita, V.T., Hellman, S. & Rosenberg, S.A. (eds) pp. 1279-1283. J.B. Lippincott: Philadelphia.

SAMSON, M.K., BAKER, L.H. & BENJAMIN, R.S. (1979). Cis-dichlorodiammine-platinium (II) in advanced soft tissue and bony sarcomas. A SWOG study. *Cancer Treat. Rep.*, **63**, 2027-2029.

SCHÜTTE, J., DOMBERNOWSKY, P., SANTORO, A., STEWARET, W., MOURIDSEN, H.T., SOMERS, R., VAN OOSTEROM, A.T., BLACKLEDGE, G., THOMAS, D. & SYLVESTER, R. (1987). Ifosfamide plus adriamycin in advanced soft tissue sarcomas. *Contrib. Oncol.*, **26**, 1659-1676.

SHORTHOUSE, A.J., SMYTH, J.F., STEEL, G.G., ELLISON, M., MILLS, J. & PECKHAM, M.J. (1980). The human tumor xenograft: a valid model in experimental chemotherapy? *Br. J. Surg.*, **67**, 715-722.

SHORTHOUSE, A.J., JONES, J.M., STEEL, G.G. & PECKHAM, M.J. (1982). Experimental combination and single agent chemotherapy in human lung xenografts. *Br. J. Cancer*, **46**, 35-44.

STEEL, G.G., COURTENAY, V.D. & PECKHAM, M.J. (1983). The response to chemotherapy of a variety of human tumor xenografts. *Br. J. Cancer*, **47**, 1-13.

STUART-HARRIS, R.C., HARPER, P.G., PARSONS, C.A., KAYE, S.B., MOONEY, C.A., GOWING, N.F. & WILTSHAW, E. (1983). High-dose alkylation therapy using ifosfamide infusion with mesna in the treatment of adult advanced soft-tissue sarcoma. *Cancer Chemother. Pharmacol.*, **11**, 69-72.

STUSCHKE, M., BUDACH, V., BAMBERG, M. & BUDACH, W. (1990). Methods for analysis of censored tumor growth delay data. *Radiat. Res.*, **122**, 172-180.

TEICHER, B.A., HERMANN, T.S., HOLDEN, S.A., WANG, Y., PFEFFER, M.R., CRAWFORD, J.W. & FREI, III, E. (1978). Tumour resistance to alkylating agents conferred by mechanisms operative only *in vivo*. *Science*, **247**, 1457-1461.

WILTSHAW, E., WESTBURY, G., HAMMER, C., MCKINNA, A. & FISHER, C. (1986). Ifosfamide plus mesna with and without adriamycin in soft tissue sarcoma. *Cancer Chemother. Pharmacol.*, **18**, 10-12.

WINOGARD, B., LOBBEZZO, M.W. & PINEDO, H.M. (1988). Proposal for the application of xenografts in screening for new anticancer agents and in selecting tumor types for phase-II clinical trials. In *Human Tumor Xenografts in Anticancer Drug Development*, Winogard, B., Peckhan, M.J. & Pinedo, H.M. (eds) pp 111-114. Springer: Berlin.